# Modern Sociology

**Published:** 1899-11-11

**Authors:** 


### Modern Sociology.

### THE EDUCATED WORKING WOMAN.

### I.—How She Lives.

Of all the subjects debated at the International Congress of Women none should, probably none did, strike such a sympathetic chord in the bosoms of the hearers as Mr. Gilbert Parker's address on the Housing of Educated Working Women. Many of those present knew the difficulties he spoke of; many had tried to solve them for themselves if not for others; but few, very few, had succeeded. In London, leaving outside altogether the industrial and commercial classes—the shop-girl and the factory-girl, who for the most part live at home—there are half a million women who work for their daily bread. A small proportion of these have private means, insufficient to maintain them, but something to fall back upon in case of emergency; more have a home to which they may retire when worn out by stress of work or ill-health. But many have nothing but their own energies between them and the workhouse—or the river. What do these earn, and how do they live?

Some, of course, earn satisfactory incomes, but the majority make from £1 to 30s. a week. If one ventures to call this a pittance there is sure to be somebody who tells us that many working-men bring up a family on an income no larger than this, which one woman enjoys for her solitary self. That is true. Yet one might fairly ask how that family is brought up, and what doles of one kind and another go to eke out the maintenance. But the real point at issue is whether an educated woman, brought up, perhaps in luxury, certainly in refinement, can adapt herself to the conditions under which these working men and their families live. Some try, for they want to economise in every way; but few can stand them. They pay the penalty of their refinement, and cannot endure the sounds, the smells, the slatternly women, the squalid children, the drunken men. Moreover, their daily work, being generally of a sedentary nature, they want decent air, and that they cannot get with such a crowd of the unwashed around them. They want quiet sleep, and that they cannot have while the British workman avails himself of his British privilege of thrashing his wife, and all the neighbours cry "murder" in chorus. The strain on her nerves is too much for the average woman. Very few can continue to live in a workman's quarter; none can do so without some loss of health, which must sooner or later affect their earning power. In short, it does not pay to try, being a round peg, to dwell in a square hole.

It is just this question of housing that most troubles the woman of whom we speak. To obtain such a lodging as she can exist in, she must, as a rule, expend half of her entire income. She needs to live in a central situation, or trains and 'buses run away with all she can save out of a cheaper rent; she needs, being a gentlewoman, who cannot make a drawing-room of the doorstep, a certain amount of privacy; and she needs a certain amount of comfort. Some of these things she is obliged to sacrifice. If she boards in a family, as used to be thought the only proper thing for a young woman living alone to do, she must sacrifice privacy, for her means will not allow of a private sitting-room. She may be tired or worried, she may have work to do; yet she must bear with the companionship of others, practically strangers to her, and often enough not congenial. Moreover, if her work causes her to keep irregular hours, it is impossible for her to dwell in a family, where a fair amount of punctuality must needs reign. So boarding is out of the question. If she tries lodgings, the room which her income will permit her to take is a meagre attic, a sort of cupboard with a window in it. Yet for this she will probably pay 8s. a week; and those myths in the creed of landladies, "attendance" and "extras," will run the rent up to 10s. Ten shillings a week out of 25s. or 30s., and food, clothing, laundry, travelling, all to be paid out of the remainder, not to speak of such real "extras" as books, stationery, recreations, or such occasional necessities as doctoring and medicine! Consider all this; consider also that while sickness is not a certainty, old age is. And what provision can our educated woman make, with all her work, for the time when she can no longer maintain herself, even in such pinched independence as she now enjoys?

Of late, blocks of buildings have been put up specially for the convenience of the class of women we refer to. These are always full, and there is usually a long list of names on the books of those who wish to enter. And yet, truth to say, many of these dwellings leave much to be desired. For one thing, many of them are too costly. One such block we remember ostentatiously announced as a charity—to the shareholders, we presume, for it has paid a 5 per cent. dividend—but those who live there must needs be among the fortunate few who have private means, or else can earn considerably over £100 a year. In others there are restrictions about latchkeys, which are, on the face of it, ridiculous. The woman who needs to be shut up at ten o'clock is not the woman who goes to these dwellings, nor is she fit to earn her living in London. Where the accommodation is really cheap, it is usually only a cubicle that can be obtained, and this is but half privacy. In one house where the terms are "inclusive," as the landladies say, there is a restriction anent the taking of both butter and jam on one piece of bread. Are women never to be allowed to get beyond the nursery stage of existence?

Perhaps Campden Houses, established by Lady Mary Feilding, form the best solution yet found for the housing of refined women at a moderate rate. The block of buildings was originally intended for workmen's dwellings, but is now reserved for ladies. You can get a tiny flat of three rooms for 12s. a week, and single rooms in proportion. A plain dinner is provided at mid-day for 8d. a head, and a little attendance is given. The fact that the building was not originally intended for its present occupants is a reason for some details not being as agreeable as they might be made; but, on the whole, these dwellings fulfil the desired end. The worst of them is that they are at Notting Hill Gate, which is not sufficiently central for many women. Yet they are always full, and there is no doubt that similar blocks, if they existed in every part of the West Central district, would be very popular. And yet these are not ideal. Cannot some better thing be devised at a cost within the means of the average working gentlewoman?

